# Analysis of the Spatial Differentiation and Promotion Potential for Agricultural Eco-Efficiency—Evidence of Pollution’s Strong Disposability

**DOI:** 10.3390/ijerph20032397

**Published:** 2023-01-29

**Authors:** Dongmei Shi, Lili Ren, Hongyu Li, Haizhen Zhang, Rufei Zhang

**Affiliations:** 1 College of Economics, Hebei GEO University, Shijiazhuang 050030, China; 2 Business School, Hebei Normal University, Shijiazhuang 050024, China; 3 The Graduate School, Woosuk University, Wanju-gun 55338, Republic of Korea

**Keywords:** agricultural carbon emissions, agricultural ecological efficiency, strong disposability, factor driver force, promotion potential

## Abstract

Agricultural eco-efficiency is an important indicator used to measure agriculture’s high-quality and sustainable development. Therefore, this paper uses the EBM-Super-ML method with strong disposability of undesired output to calculate Chinese agricultural eco-efficiency and uses a geographical detector to measure the driving force of the factor. The research conclusions are mainly reflected in three aspects. Firstly, from the perspective of eco-efficiency changes, the overall mean value of agricultural eco-efficiency increased by 3.5%, and the regional heterogeneity is significant, with the fastest growth in the eastern region. Secondly, the results of driving force analysis show that the main driving factors of agricultural eco-efficiency divergence are capital inputs, total carbon emissions, labor inputs, agricultural film residues, fertilizer use, and pesticide residues, with driving forces of 0.43, 0.37, 0.34, 0.31, 0.28, and 0.20, respectively. Finally, from the perspective of eco-efficiency improvement potential, the mean value of output improvement potential is 5%, and the input factor is 7%. Among the non-desired outputs, the reduction rate of agricultural films can reach 40%. Among the input factors, labor input has the highest potential for intensive use, while agricultural machinery has a negative effect. Therefore, strengthening the development of the agricultural service industry is of great significance to improve the utilization rate of mechanical equipment and reduce the undesired output of agriculture.

## 1. Introduction

Since the reform and opening in 1978, the Chinese agricultural economy has developed rapidly. The total output of grain, meat, and aquatic products accounted for about 20%, 25%, and 33% of the corresponding supply in the world in 2017, but the application amount of chemical fertilizer, mulching films, and pesticide increased by 6.6 times, 5.3 times and 2.3 times, respectively [1]. According to the First National Pollution Source Census Bulletin (2010), the three main pollutants, chemical oxygen demand (COD), total nitrogen (TN), and total phosphorus (TP) emitted by agricultural pollution sources in China reached 1324.09, 270.46, and 284.7 thousand tons, respectively, accounting for 43.7%, 57.2%, and 67.3% of the total. Agricultural pollution has become the largest source of pollution. The huge pollution challenges brought by the development of the agricultural economy need to be solved urgently, which is also an important research goal of this paper.

The agricultural production mode depends on the high input of production factors, which leads to the aggravation of agricultural non-point source pollution. [2]. In addition, the utilization rate of agricultural resources in China is relatively low; the utilization rates of chemical fertilizer and pesticides were 37.8% and 38.8% [3], respectively. The total utilization rate of livestock and poultry manure was about 60%, and the recovery rate of the agricultural film was less than 2/3 in 2018. It shows that China’s agricultural growth mainly depends on the intensive input of production factors. For this reason, the government has paid more attention to the development of ecological agriculture [4] and issued a series of policies [5] to provide strong support for the healthy and sustainable development of the national economy and society.

Many research studies have quantified the performance of agricultural eco-efficiency [6]. A branch of related research is the concept of agricultural eco-efficiency. The sustainability of agriculture can be well represented by “eco-efficiency”, which is defined in agricultural research as the ratio of the economic value added by agricultural production to the environmental impact [7], and can also be understood as sustainable intensification. Agro-ecological efficiency reveals changes in the interactions between agro-ecosystems and man–land systems. Measuring eco-efficiency provides important information for policy-makers to formulate policies focusing on sustainable management and efficient use of natural resources in the agricultural sector, providing important indicators for decision-makers to formulate economic development strategies [8].

There are also related studies examining the performance of agricultural eco-efficiency. The existing studies mainly adopted the ratio method, life cycle assessment (LCA) [9], ecological footprint method, stochastic frontier analysis (SFA) [6] and data envelopment analysis (DEA) [1,6]. SFA and DEA are the two most used methods [9], among which the most used DEA indices include the super-efficiency model, directional distance function (SBM) [10], mixed distance function (EBM) [11], dynamic panel data efficiency, and so on [12].

The third is the study of efficiency evaluation. The existing literature mainly discusses the indicators of agricultural resource allocation efficiency, agricultural joint production efficiency, evolution characteristics of efficiency and other aspects [13]. The analysis results showed that the characteristics of agricultural eco-efficiency in China were unbalanced. On the one hand, due to differences in economic development levels and resource endowments, there are obvious regional differences and spatial agglomerations in China’s agricultural ecological efficiency [13]. On the other hand, the main reasons for the low efficiency of AEE are excessive agricultural machinery, land sown area and agricultural carbon emissions [13].

The fourth is the driver of efficiency. The existing literature mainly uses the geographic detector method to analyze the driving force of efficiency, and the analysis results show that the widely used regional stratification methods have insufficient explanation for the spatial differentiation of agricultural ecological efficiency. The hierarchical method of factor efficiency has a high degree of explanation for spatial differentiation. Excessive agricultural machinery, land input and excessive agricultural carbon emissions are important reasons for the low efficiency of agricultural ecology [7].

In conclusion, the existing literature mainly focuses on measuring and analyzing the spatial–temporal evolution characteristics of agricultural eco-efficiency. It rarely explores the causes of agricultural eco-efficiency (AEE) loss by combining the slack of agricultural input factors and output insufficiency [14]. Therefore, we use the Strong-Disposability-EBM Super-Efficiency Undesirable Malmquist Productivity Index to estimate the AEE, probe feature factor-driving forces, and improve potential in China. The main research contents are as follows. Firstly, we establish an EBM-super-efficiency ML model from the strong disposability perspective of agricultural pollution to study the spatial and temporal differences of AEE and its improvement targets, which provide a theoretical basis for analyzing agricultural eco-efficiency. Secondly, we explore the factor-driving forces and the interaction of significant factors that affect the heterogeneity of AEE. Thirdly, our work analyzes the inefficiency of input and output factors from two perspectives of slackness and redundancy to explore the improvement potential of AEE in China, which has important theoretical and practical implications for promoting sustainable agricultural development.

This article is characterized by three aspects. The first is the measurement of carbon emission indicators. This paper comprehensively considers the shortcomings of the existing literature in calculating agricultural carbon emissions, and selects four main emission sources, including land ploughing, modern agricultural production, and methane (aquaculture and cultivation). Land tillage before farming will lead to the release of carbon dioxide adsorbed in the soil again. The use of a large number of mechanical and fossil products (fertilizers, pesticides, etc.) in modern agricultural production will directly produce greenhouse gases. In addition, this paper measures the carbon emissions of major livestock and poultry breeding in China and the carbon emissions of early rice and late rice. The second is the method of measuring agricultural ecological efficiency. Based on the theory of joint production, there are not only expected outputs in the production process of agriculture, but also undesired outputs such as non-point source pollution and carbon emissions, which are different from the non-expected outputs of industry, which have strong disposability characteristics, so the paper chooses the strong disposability EBM-super-efficiency model. The ML model selects a fixed reference method with a base period of 2009, which makes the measurement results more comparable. The third is an in-depth analysis of the calculation results. The analysis of model measurement results is not in-depth enough, the analysis of inefficiency is less, and there is little analysis of the improvement of elements, so the analysis of this paper has important reference significance.

## 2. Method

Agricultural eco-efficiency is the ratio of the economic value added by agricultural production to the consequences of environmental impact [15]. DEA method is the most commonly used for AEE evaluation [16]. Compared to the SFA model, the DEA method ignores the influence of random errors and can overcome the impact of non-technical factors on the frontier production function [17]. DEA method has some advantages, including simultaneous processing of multiple input–output elements and nonparametric processing of effective boundaries. Obtaining the current output level at a lower input level is more conducive to achieving the goal of sustainable agricultural development [18]. This paper controlled the state of economies of scale, orientation, disposability of elements, and production frontier functions. The work uses the EBM-super-ML index to measure the AEE of various regions in China and further analyze driving forces of spatial differentiation and the improvement potential for AEE.

### 2.1. EBM Super-Efficiency Model

Tone and Tsutsui proposed the EBM (epsilon-based measure) model. It is a hybrid model that includes two types of distance functions: radial and SBM. The non-point source pollution and carbon emission in agricultural production are counted as unexpected outputs.

Evaluation of agricultural eco-efficiency should consider not only the growth of agricultural economic benefits but also undesirable outputs (bad outputs) such as non-point source pollution and carbon emissions generated during agricultural production. Unlike industrial production, these undesirable outputs are characterized by strong disposability. Therefore, the study selects the strong disposability non-expected output EBM distance function model. The model’s projection direction of the evaluated DMU is to increase good output and reduce bad output.

Referring to the setting of the model by Wang, the super-efficiency EBM model has four inputs, one expected output, and four unexpected outputs. In the planning formula, max represents the strong effective frontier of the disposability of undesirable output. Thirty provinces are recorded as decision-making units as $x_{ij}$, in period *t* (*t* = 1, …, *T*). There are *k* (*k* = 1, …, 30) DMUs, each decision-making unit has *m* input *X* = ($x_{1},x_{2},\cdots ,x_{m}$)$\in R_{+}^{m}$, *Y_r_* = (*r* = 1, 2, …, *n*)$\in R_{+}^{n}$ and *J* non-expected output *Y_j_* = $(j=1,\ldots ,j)\;\in \;R_{+}^{j}$, *X* = {*x_ij_*} ∈ *RM* × *N*, *Y* = {$y_{ij}$*} ∈ RM × N*, and *X* > 0, *Y* > 0, respectively, are input and output matrices.

The production possibility set of the model indicates that under the given input condition, the good output can be reduced or the bad output can be increased. This production possibility set means that bad output can be increased indefinitely with a given input, which means strong disposability of bad output. Here, the model is called the strong disposable non-expected output EBM distance function model. The difference between strong disposability and weak disposability in linear programming is that strong disposability uses the inequality sign and weak disposability uses the equal sign. The production possibilities set and model settings are as follows.

(1) $$\rho^{*}=max\frac{\theta -\varepsilon_{x}\sum_{i=1}^{4}\frac{W_{i}^{-}S_{i}^{-}}{x_{ik}}}{\varphi +\varepsilon_{y}\sum_{r=1}\frac{W_{r}^{+}S_{r}^{+}}{y_{rk}}+\varepsilon_{b}\sum_{p=1}^{j}\frac{W_{p}^{b_{-}}S_{p}^{b_{-}}}{b_{pk}}}$$

$$s.t.\;\sum_{j=1,\;j\neq jo}^{k}x_{ij}\lambda_{j}+S_{i}^{-}=\theta X_{ik},i=1,2,3,4\;\sum_{j=1,\;j\neq jo}^{k}y_{rj}\lambda_{j}-S_{r}^{+}=\varphi y_{rk},r=1\;\sum_{p=1}^{2}b_{pj}\lambda_{j}+S_{p}^{b_{-}}=\varphi b_{pk},p=1,2,3,4$$

$$\lambda_{j}\geq 0,S_{i}^{-},S_{r}^{+},S_{p}^{b_{-}}\geq 0$$

The production possibilities set:

$$S=\{\left(x,y\right):x\geq X\lambda ,\;y\leq Y\lambda ,\;b\geq B\lambda \}$$

where ρ* is the best efficiency under the condition of constant return to scale. θ is the planning parameter of the radial part. $\varepsilon_{x},\;\varepsilon_{y}$ is key parameter. Satisfy 0$\;\leq \varepsilon_{x},\varepsilon_{y}\leq 1.\;W_{i}^{-}$ is the importance of input indicators, it meets $\overset{4}{\underset{i=1}{\sum }}W_{i}^{-}$ = 1; $x_{ik}$ and $y_{rk}$ are the *i* inputs and the r outputs of decision-making of DMUk. $S_{i}^{-}$ is the relaxation of input element *i*. φ is the output expansion ratio. $S_{r}^{+}$ is the relaxation variable of the expected output of class *r*. $S_{p}^{b_{-}}$ is the relaxation variable of *p*-type unexpected output. $W_{r}^{+},W_{p}^{b_{-}}$ is the weight of both indices. $b_{pk}$ is the *p* unexpected output of DMUk, *q* is the number of unexpected outputs, *j* is the DMU, $\lambda_{j}$ is the linear of combination coefficient. $j_{0}$ represents the super-efficiency value of DM$U_{j}$ on the new effective frontier excluding DM$U_{jo}$ when the commented decision unit is DM$U_{jo}$.

The work used the EBM function to calculate agricultural ecological efficiency, taking into account the strong disposability of unexpected outputs such as land non-point source pollution and carbon emission, and set the intensive potential of expected output and unexpected output to 1:1; more consideration is given to the emission reduction potential of unexpected output, which is different from the model setting in most literatures, and the conclusions are quite different.

### 2.2. ML Index

The Malmquist index is usually used to analyze the panel data of observed values at multiple time points. Färe first used the DEA method to calculate the Malmquist index (MI). Further, it is decomposed into technical efficiency change (EC) and technical change (TC), commonly used to analyze productivity changes and the effect of technological efficiency and technological progress on productivity change. Chung introduced the directional distance function into the Malmquist index to deal with unexpected output, called the Malmquist–Luenberger (ML) index [19]. The core is to solve the problem of unexpected output. The Fixed Malmquist index takes the single-phase front of a fixed period as the reference front for calculating MI (*t* − 1, *t*) in each period [19]. The MI and its decomposition efficiency model are as follows:

$$M_{g}^{t}\left(x_{k}^{t},y_{k}^{t},x_{k}^{t-1},y_{k}^{t-1}\right)=\frac{D_{k}^{g}\left(x_{k}^{t},y_{k}^{t}\right)}{D_{k}^{g}\left(x_{k}^{t-1},y_{k}^{t-1}\right)}$$

$$EC=\frac{D_{k}^{t}\left(x_{k}^{t},y_{k}^{t}\right)}{\;D_{k}^{t-1}\left(x_{k}^{t-1},y_{k}^{t-1}\right)}$$

$$TC=\frac{D_{k}^{g}\left(x_{k}^{t},y_{k}^{t}\right)}{D_{k}^{t}\left(x_{k}^{t},y_{k}^{t}\right)}\cdot \frac{D_{k}^{t-1}\left(D_{k}^{t-1},y_{k}^{t-1}\right)}{D_{k}^{g}\left(x_{k}^{t-1},y_{k}^{t-1}\right)}$$

(2) $$\mathrm{MI}=\frac{D_{k}^{t}\left(x_{k}^{t},y_{k}^{t}\right)}{D_{k}^{t-1}\left(x_{k}^{t-1},y_{k}^{t-1}\right)}\cdot \left(\frac{D_{k}^{g}\left(x_{k}^{t},y_{k}^{t}\right)}{D_{k}^{t}\left(x_{k}^{t},y_{k}^{t}\right)}\cdot \frac{D_{k}^{t-1}\left(D_{k}^{t-1},y_{k}^{t-1}\right)}{D_{k}^{g}\left(x_{k}^{t-1},y_{k}^{t-1}\right)}\right)=EC\cdot TC$$

where *x* and *y* are the input index and output index, respectively. $x_{k}^{t}$ represents the input vector of the *k* region in year *t*. $y_{k}^{t}$ represents the vector of agricultural output for the *k* region in year *t*; *θ* represents the technical efficiency index; $D_{k}^{t}$ represents the distance function of the production point in year t in reference to the technology *Tt* in year *t*.

### 2.3. Geographical Detector

The geographic detector method can measure the spatial heterogeneity of variables. The detection results mainly include two aspects: one is to test the most considerable stratified heterogeneity of a single variable and driving factors causing the spatial differentiation. The other is to test the coupling of the spatial distribution of two variables to detect possible causal relationships between two variables [19]. The methods of differentiation and factor detection, and interaction detection are as follows:

Spatial Difference and Factor Detection. This method uses the power determinant value indicator to measure the degree to which the spatial differentiation of the dependent variable is affected by the spatial differentiation of the independent variables [4]. By introducing the power determinant value *q*(*X*|{*h*}), we explore the driving factors of AEE. The larger the corresponding *q* value, the greater the explanation degree of the spatial differentiation of the dependent variable. The study sets the AEE as dependent variable *Y* and the driving factor *X* = {*Xh*} (*h* = 1, 2, …, *l*; *l* is the number of partitions of the factor). *Xh* represents the different partitions of factor *X*, the dependent variable *Y*, and the factor. The *X* layers are superimposed to represent the determining force of factor *X* on the dependent variable *Y*. The *q* value can be represented by the Formula (3).

(3) $$q=1-\frac{\sum_{h}^{L}N_{h}{\sigma_{h}}^{2}}{N\sigma^{2}}=1-\frac{SSW}{SST};SSW=\sum_{h}^{L}N_{h}{\sigma_{h}}^{2},SST=N\sigma^{2}$$

where *N* is the number of units in the entire study area, *N_h_* is the number of units contained in the *h*-th subregion of factor *X*, $\sigma^{2}$ represents the variance of *Y* values in the entire study area, and ${\sigma_{h}}^{2}$ is the variance of *Y* in the *h*-th subregion of the driving factor *X*. *SSW* and *SST* are the sum of the variances of the sampling units in each sub-area and the total variance of the sampling units in the whole area, respectively. *q* indicates that the driving factor *X* explains 100 × *q*% of the spatial distribution of *Y*, and the value range is [0, 1].

To compare whether the cumulative variance of each subregion is significantly different from that of the entire study region, the *F* statistic is shown in Equation (4).

(4) $$\begin{array}{cc} F & \;=\frac{N-L}{L-1}\frac{q}{1-q}∼F\left(L-1,N-L;\lambda \right)\\ \lambda & =\frac{1}{\sigma^{2}}[\sum_{h=1}^{t}Y_{h}^{2}-\frac{1}{N}\left(\sum_{h=1}^{t}\sqrt{N_{h}}{\overset{ˉ}{Y}}_{h}{)}^{2}\right] \end{array}$$

where, λ is the non-centrality parameter, ${\overset{ˉ}{Y}}_{h}$ is the mean of the subrange.

Interaction Detection. Interaction detection is used to measure the degree of interpretation of the dependent variable Y when there is an interaction between independent variables or whether the effects of these independent variables on the dependent variable Y are independent of each other. The detection results represent that risk factors $X_{1}$ and $X_{2}$ (and more X) interact with the response variable Y. The types of interactions between the two independent variables on the dependent variable are shown in Table 1.

## 3. Variables and Data

### 3.1. Input and Output Indicators

The optimal agricultural production efficiency must consider both agricultural production increase and emission control [20]. Most literature believes that the selection of output variables should include expected and unexpected output [21]. From the C-D production function perspective, input factors mainly include labor force, land, capital, and technology [22]. The expected output of agriculture consists of the gross output value of agriculture, forestry, animal husbandry, and fishery, which measures the economic benefits and overall results [23]. Unexpected agricultural production mainly comes from the excessive input or inefficient utilization of some production factors. The statistics of agricultural pollution mainly include COD and TN and P emissions [24]. Therefore, as Table 2 shows, the unexpected output in this paper comprises fertilizer residue, pesticide residue, agricultural film residue, and carbon emission of agricultural products.

### 3.2. Calculation Method of Carbon Emission Index

Agricultural greenhouse gases include CH_4_ and N_2_O from agricultural land, animal intestinal fermentation, and manure management [25]. According to the unit survey and assessment method, agricultural carbon emissions mainly come from petrochemical products such as chemical fertilizers and pesticides, agricultural irrigation, tillage, agricultural machinery power, and methane emissions from animal husbandry and breeding production [26]. The work calculates agricultural carbon emissions from three aspects: input of agricultural materials [27], carbon emissions from crop planting, and animal husbandry. The calculation formula is as follows:

(5) $$ACE=\sum_{i=1}^{n}E_{i}\times \delta_{i}=\sum_{i=1}^{n}(C+CH_{4crop}+N_{2}O_{crop}+N_{1i}+N_{2i})$$

(6) $$C=\sum_{i=1}^{6}C_{i}=\sum_{i=1}^{6}X_{i}\cdot h_{i}$$

(7) $$CH_{4crop}=\sum_{i=1}^{n}S_{i}\times \alpha_{i}$$

(8) $$N_{2}O_{crop}=\sum_{i=1}^{n}S_{i}\times \beta_{i}+P_{i}\times \gamma_{i}+Q_{i}\times \delta_{i}$$

(9) $$N_{1i}=Days\_alive_{i}\times \frac{M_{i}}{365}$$

(10) $$N_{2i}=C_{i}+C_{it-1}/2$$

In formula, *ACE* is total hydrocarbon emission, $E_{i}$ is the consumption of class ith energy. $\delta_{i}$ is the carbon emission coefficient of class ith energy.

In Formula (6), $C_{i}$ is the carbon emission of agricultural supplies, $X_{i}$ is the amount of each carbon emission source, $h_{i}$ is the carbon emission coefficient of each carbon emission source.

In Formulas (7) and (8): $CH_{4crop}$ is the total annual methane emission of the planting industry. $S_{i}$ is the sowing area of the crop, $\alpha_{i}$ is the methane emission coefficient per unit area of the crop. $N_{2}O_{crop}$ is the annual emission of nitrous oxide from the planting industry, $\beta_{i}$ is the yearly emission background flux of nitrous oxide per unit area of the crop. *Pi* is the annual total amount of nitrogen fertilizer applied to the crop, and $\gamma_{i}$ is the nitrous oxide emission coefficient of the crop. $Q_{i}$ is the total annual application amount of compound fertilizer for the crop, $\delta_{i}$ is the nitrous oxide emission coefficient of compound fertilizer for the crop.

In Formulas (9) and (10), $N_{1i}$ is the annual average feeding number of livestock with a slaughter rate greater than 1. $M_{i}$ is the annual slaughter number of ith livestock. $N_{2i}$ is the average yearly feeding number of livestock with a slaughter rate less than 1. $C_{i}$, $C_{\left(it-1\right)}$ represent the year-end stock of livestock.

### 3.3. Data Sources

Referring to most kinds of literature, the original data of model indicators come from yearbooks, such as the Chinese Rural Statistical Yearbook, the Chinese Agricultural Economic Yearbook, and the annual statistical report of Chinese rural management, from 2009 to 2019. According to the availability and integrity of data, we chose generalized agriculture as the research object, including 30 provinces and autonomous regions of the Chinese mainland, and excluded incomplete data from Hong Kong, Macao, Taiwan, and Tibet. The provincial administrative region division method was approved by the State Council in 2000 and divided the 30 local regions into East, Central, and West. The missing data of individual samples are processed by the interpolation method. It was adjusted to the constant price-output value in 2009 to eliminate inflation.

### 3.4. Descriptive Statistics

**Figure 1:**
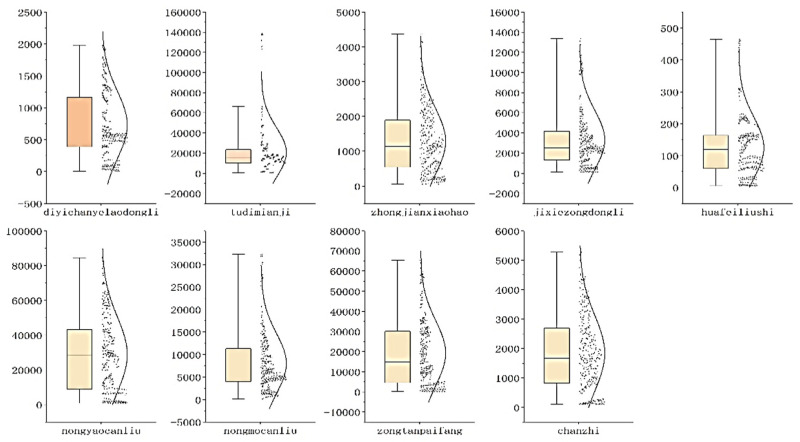
Box chart of input and output indicators from 2009 to 2018.

As Figure 1 shows, land area indicators are most concentrated, while pesticide residue indicators are scattered. To understand the relative difference between the indicators, we measure the standard deviation to reflect the fluctuation of the unit means in Table 3. Among them, the coefficient of variation of the land area is the largest at 1.19. It shows that the land input of the primary industry varies significantly between different provinces. The coefficient of variation of each indicator is relatively stable, around 0.8, indicating differences on the one hand, while the fluctuations are similar.

**Figure 2:**
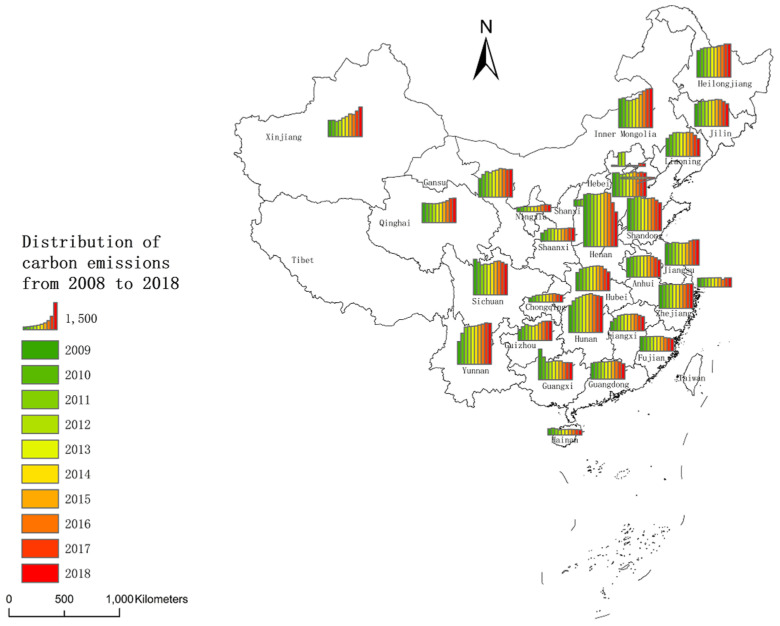
Regional distribution of mean CO_2_ emissions from 2009 to 2018.

Distribution of CO_2_. As Figure 2 shows, from the regional distribution of average agricultural CO_2_ emissions, Beijing, Tianjin, Hainan, Ningxia and Chongqing have lower emissions, while Henan, Hunan, Sichuan, Yunnan, Inner Mongolia and Hebei have high emissions. The emission values of Jilin, Beijing, Tianjin, Henan, Shandong and Fujian are in a downward trend, and the emission values of Xinjiang, Qinghai, Heilongjiang, Yunnan, Inner Mongolia, Guizhou and Jiangsu are increasing.

## 4. The AEE Calculation Results

The paper used Maxdea8.0 to measure the agricultural ecological efficiency in China, and analyzed the results of the time and space distribution. The time distribution trend is analyzed from the population mean and kernel density distribution. The spatial distribution trend is analyzed from regional mean and cluster distribution.

### 4.1. Timing Characteristics of AEE

**Figure 3:**
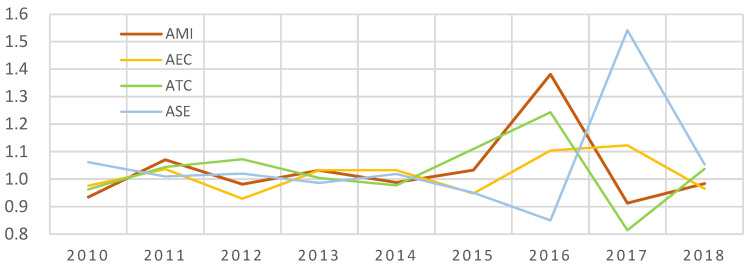
National Average Fluctuation of MI, EC, SE, and TC from 2010 to 2018. Note: A is the representative mean value. MI is comprehensive efficiency, EC is technical efficiency, TC is technical progress, and SE is scale efficiency.

According to the model estimation result of the ML index, the MI is comprehensive efficiency and mainly refers to AEE in the paper. Its decomposition efficiency includes EC, TC, and SE. Among them, the EC is technical efficiency, TC is technical progress, and SE is scale efficiency. As shown in Figure 3, the mean value of AEE was more significant than one from 2010 to 2018, and the overall growth was in a state of development. Among them, the fluctuations in 2010–2015 were relatively small and relatively large in 2016–2018. The fluctuation range of EC is relatively tiny, and the SE average fluctuates wildly. The average value of MI is affected by TC and declined from 2017 to 2018.

### 4.2. The AEE Kernel Density Distribution

**Figure 4:**
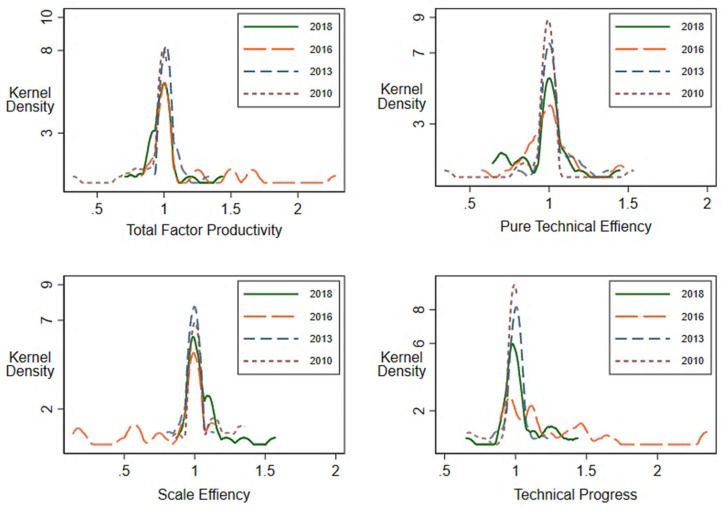
MI, EC, SE, and TC kernel density distribution in representative years.

As shown in Figure 4, in the representative years from 2010 to 2018, the wave peak’s MI, EC, SE position is unchanged, and in some years with small wave peaks, a high–high and low–low agglomeration phenomenon appears. The peak position of the TC wave is unstable, and the low–low aggregation is more evident in 2016 and 2018. From the kurtosis point of view, the AEE was relatively concentrated in 2010, and inefficient and ultra-efficient units accounted for a relatively low value. The peak value in 2016 was the smallest, with multiple peaks, indicating that the efficiency value had multiple convergence points. Numerical values are relatively scattered; there is no significant change in the width of kurtosis during the study period, indicating that the differences between provinces did not change significantly.

### 4.3. The AEE Spatial Distribution

**Figure 5:**
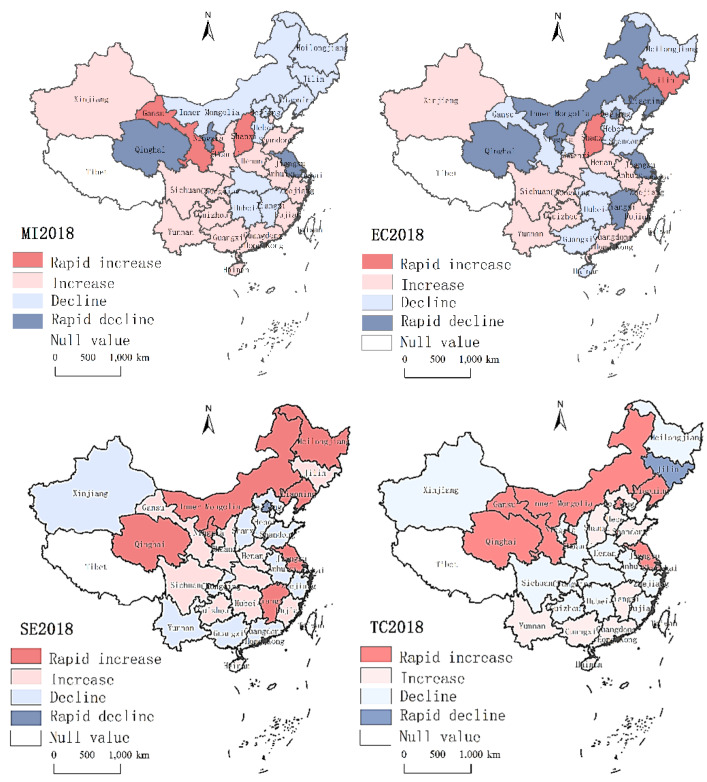
Spatial geometric mean distribution of AEE in 2018.

The mean efficiency value can reflect the sample’s overall trend, and different regions have different characteristics [28]. We analyzed the Chinese spatial pattern AEE in mean scale and agglomeration distribution aspects [29]. According to the fluctuation range of the efficiency value, we divided China’s agricultural total factor productivity into four types: rapid growth (<1.2), increase (1–1.2), decrease (0.8–1), and rapid decline (>0.8). As shown in Figure 5, the trend of aggregation of efficiency values is relatively apparent, and the efficient development of different regions is different. In 2018, the provinces with lower MI in the western region saw rapid growth in SE and TC, while EC fell rapidly, and in-depth analysis is needed. The eastern region is economically developed, and the MI of most provinces are in a state of growth, but the SE and TC values are low; the scale efficiency SE in the central region is better than others, and the TC is decreasing. From the average point of view, the provinces in the high-efficiency group are located in the eastern and western regions. In contrast, some provinces in the northeast, west, and central regions are mainly in the middle and low-efficiency groups, consistent with the conclusions of Fang [30].

## 5. Spatial Stratified Heterogeneity

The analysis results of agricultural eco-efficiency showed significant spatio-temporal differences in agricultural eco-efficiency in different regions of China. Therefore, we further used geographic detectors to identify the leading factors affecting the spatial differentiation of agricultural eco-efficiency and their interactions to explore the main factors affecting the spatio-temporal differentiation of agricultural eco-efficiency.

### 5.1. Index Setting and Data Processing

Concerning the relevant literature, the input–output factors in agricultural production in each province were taken as driving factors to investigate the driving factors and forces of spatial differentiation of agricultural eco-efficiency [6,13]. The hierarchical processing was carried out and discretized into type variables, and each driving factor’s single-factor influence degree and multi-factor interaction on AEE were measured. The driver setting and its discrete classification results are shown in Table 4.

### 5.2. Factor Driver Force

This paper calculates the driving force of each factor on the spatial differentiation of AEE from 2009 to 2018. So that the determinative power of each driving factor on the spatial differentiation of AEE can be compared over time, we select the result of 2010, 2012, 2014, 2016, and 2018. The calculation results are shown in Table 5. 

Based on the horizontal factor comparison results, labor input and capital investment have the most prominent driving force effect on AEE. Total carbon emissions, pesticide residues, fertilizer use, pesticide use, and agricultural film residues have significant driving effects. Detailed conclusions are as follows.

First, the spatial driving force of labor input is generally on the rise. In 2010, the explanatory power of labor input on the spatial differentiation of AEE was 0.334, which rose to 0.431 in 2018. It shows that the ability of labor input to explain the spatial differentiation of agro-ecological efficiency is getting stronger and stronger. Second, the value of the spatial driving force of capital investment is on the rise, rising from 0.384 in 2012 to 0.553 in 2018, which indicates that in 2018, the ability of capital investment to explain the spatial differentiation of China’s agricultural ecological efficiency reached 55.3%. Thirdly, the spatial driving force values of chemical fertilizer and pesticide use show an upward trend year by year, and the explanatory power was 33.9% and 26.0% in 2018. Fourth, the spatial driving force of pesticide residues passed the significance test since 2016. In 2018, the spatial driving force was 26%, increasing the spatial differentiation of agricultural ecological efficiency. Fifth, the agricultural film residue passed the significance test in 2018, and the driving force value was 0.479, which significantly improved compared with 0.305 in 2010. This change shows that the impact of agricultural film residue on the spatial difference of agricultural eco-efficiency is becoming more and more significant. Sixth, the spatial driving force value of total carbon emissions passed the significance test from 2010 to 2012, and the spatial driving force value in 2012 was 0.673, but it has not passed the significance test since 2014. The impact shows an inverted “U” trend, which is closely related to the implementation of the country’s energy conservation and emission reduction policies and sustainable development strategies.

### 5.3. Factor Interaction Effect

Through factor detection analysis, it was found that there were six factors with an average driving force exceeding 20%. Therefore, this paper analyzes the interaction of factors affecting the spatial differentiation of agricultural eco-efficiency. The comprehensive effect of the two factors will improve the explanatory power of the spatial differentiation of agricultural ecological efficiency. Even-numbered years are used as research samples for analysis, and the detection results, as Table 6 shows.

From the detection results in Table 4, it can be seen that during the sample period, the spatial driving force values obtained from the interaction of driving factors all showed different degrees of improvement. From the perspective of action types, 83% of the interaction types among the dominant factors are a non-linear enhancement, and the non-linear effects of each factor in the observed sample gradually weaken. The two-factor effect has gradually increased and significantly increased in 2018. In the observation sample, the factor effects of labor input and fertilizer use exceeded 0.8. In 2018, there were two-factor effects, indicating that the production method using manual operations will use more fertilizers. In 2010, the non-linear interaction between labor and agricultural film residues was 0.99, and the non-linear interaction with carbon emissions was 0.89. The non-linear interaction between agricultural film residues and total carbon emissions was 0.89. In 2012, the non-linear interaction between capital investment and agricultural film residues was 0.96. In 2014, the non-linear effect of capital investment and agricultural film residue was 0.96, and the non-linear effect of labor and fertilizer was 0.89. In 2016, the non-linear effect of labor, fertilizer, and carbon emissions was 0.86, and the non-linear effect of capital and agricultural film residue was 0.88. In 2018, the forces of labor and chemical fertilizers, agricultural film residues, and capital and agricultural film residues all exceeded 0.85.

## 6. The Improvement Potential of AEE

Modern agricultural production improves economic benefits while destroying the agricultural ecological environment and efficiency. It is reflected in the redundant input of agricultural production factors and insufficient agricultural output. The degree of AEE inefficiency is the potential improvement of agricultural ecological efficiency. The improvement approach includes two aspects. One is to achieve the maximum agricultural output under the premise of a given factor input and ecological environment impact; the second is to use fewer agricultural production factors to achieve a given output. We analyzed the situation of agro-ecological inefficiency from two aspects; one is the overall distribution of ecological inefficiency, and the other is the redundancy and relaxation of production factors. Therefore, the paper analyzed the situation of agro-ecological inefficiency from two aspects; one is the overall distribution of ecological inefficiency, and the other is the redundancy and relaxation of production factors.

### 6.1. Efficiency Decomposition

This section uses the factor inefficiency decomposition formula based on DEA to investigate the improvement potential of agricultural eco-efficiency in China by region. This paper considered strong disposability characteristics of non-desired outputs such as land non-point source pollution and carbon emissions when measuring agricultural eco-efficiency. The non-directed EBM function was adopted to set the intensive potential expected, and the non-expected outputs as 1:1. The epsilon-based measure methods were selected for calculation, and the emission reduction potential of non-expected output was considered more. The utilization of each input factor in agricultural production is evaluated by measuring the inefficiency and redundancy rate of input factors and output. The higher the redundancy rate, the lower the input–output efficiency and the greater the intensive utilization potential of input factors.

According to the measurement principle of agricultural eco-efficiency, the projection value (target value) is the projection of the evaluated DMU on the leading edge. The relative gap between the current value and the projection value represents the inefficiency of each input and output of the DMU. The improvement value of an inefficient DMU includes two parts: proportional movement and slack movement. The calculation formula is projection value = original value + proportional improvement value + slack movement. Combined with Cooper’s decomposition idea, the efficiency decomposition formula and inefficiency decomposition formula of input–output elements are as follows:

(11) $$e_{i}=\frac{x_{ik}^{T}-s_{i}^{-}}{x_{ik}^{T}},\;e_{r}=\frac{y_{rk}^{g,T}}{y_{rk}^{g,T}+s_{r}^{+}}\;,\;e_{q}=\frac{y_{qk}^{b,T}-s_{q}^{-}}{y_{qk}^{b,T}}.$$

(12) $$ie_{i}=\frac{1}{2m}\;\frac{s_{i}^{-}}{x_{ik}^{T}}\;,\;ie_{r}=\frac{1}{2\left(s_{1}+s_{2}\right)}\;\frac{s_{r}^{+}}{y_{ik}^{g,T}}\;,\;ie_{q}=\frac{1}{2\left(s_{1}+s_{2}\right)}\;\frac{s_{q}^{-}}{y_{qk}^{b,T}}.$$

where $s_{i}^{-}$, $s_{r}^{+}$, and $s_{q}^{-}$ are relaxation variables of input, expected output and unexpected output, respectively; $e_{i}$, $e_{r}$, and $e_{q}$ represent the efficiency decomposition formula of input factors, expected output and unexpected output, respectively, and $ie_{i}$, $ie_{r}$, and $ie_{q}$ represent the inefficiency decomposition formula, respectively. The efficiency decomposition formula obtains the efficiency level of each element, and the inefficiency decomposition formula obtains the decomposition of the total inefficiency, reflecting the promotion potential of each element to agricultural ecological efficiency.

### 6.2. The Overall Situation of AEE Inefficiency

**Figure 6:**
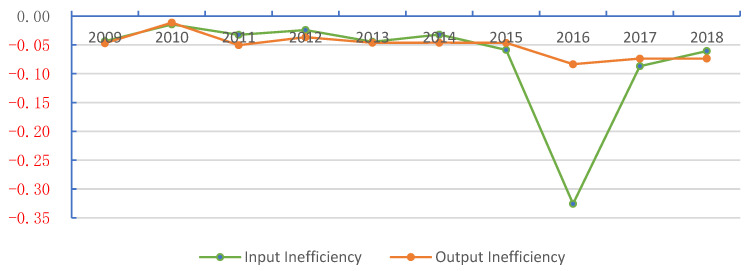
Overall mean value of input–output inefficiency from 2009 to 2018.

Inefficiency is the main factor in measuring the improvement potential of AEE. As shown in Figure 6, from 2009 to 2018, the overall ratio of agro-ecological inefficiency was 0.7333. The average agricultural input factor inefficiency value was 0.0723, and the average output inefficiency was 0.0515. In comparison, the proportion of input factors was unbalanced. It is the main reason for China’s agricultural ecological efficiency decline. The inefficiency value of input factors fluctuates wildly, reaching the maximum inefficiency value of 0.3257 in 2016. The inefficiency value of output factors fluctuates little. It is generally on a downward trend, which reflects that China attaches importance to the high-quality development of the agricultural industry and the protection of AEE in recent years. The efficiency measurement results show that the inefficient regions are mainly located in the western region, including Gansu (0.7569), Shanxi (0.8944), Jilin (0.9620), and Heilongjiang (0.9932).

### 6.3. Factor Redundancy

The unreasonable factor configuration structure is an important reason for inefficiency. Through the redundant analysis of input–output factors, it is of great significance to further explore the improvement potential of AEE.

**Figure 7:**
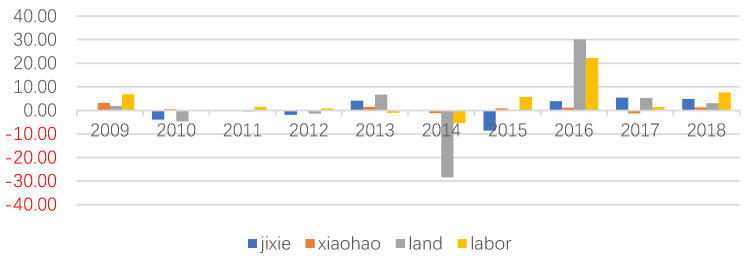
Intensive utilization potential of AEE input factors from 2009 to 2018.

Redundancy of input factors. As shown in Figure 7, from 2009 to 2018, the average intensive utilization potential of the primary industry’s labor force, land, intermediate consumption, and total mechanical power was 3.89%, 1.18%, 0.54%, and 0.36%. It indicates that the potential for intensive utilization of the labor force in agricultural production is significant, with the redundancy ratio exceeding 20% in 2016. The second is the land, with the redundancy ratio exceeding 30% in 2016. It is necessary to reduce land input or improve the intensive use of other input factors to improve the land output rate [31].

**Figure 8:**
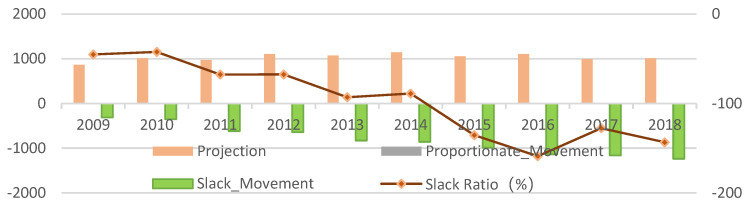
Intensification potential of national average output from 2009 to 2018.

Redundancy of expected output. As shown in Figure 8, from 2009 to 2018, considering carbon emissions and non-point source pollution of petrochemical supplies, the mean value of the target output value changed slightly, rising first and then falling. In 2018, it was somewhat higher than in 2009, with a growth rate of 8.31%. The average real output value showed a yearly rise, with a growth rate of 91.71%. As shown in the figure, the slack of the real output value keeps increasing, and the actual output value is much higher than the projection value, with the adjustment range rising from 45.2% to 158.89%. It is necessary to adjust the input proportion of production factors appropriately, improve the quality of agricultural products [32], and transition from quantity growth to quality improvement.

Redundancy of unexpected output. Unexpected output indicators include carbon emissions and non-point source pollution of petrochemical products.

**Figure 9:**
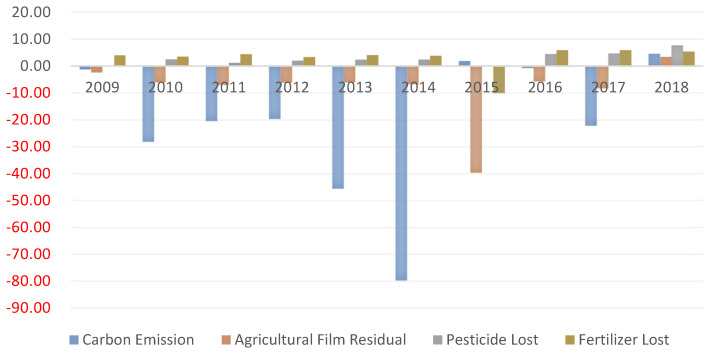
Redundancy of unexpected output from 2009 to 2018.

Total carbon emissions. As shown in Figure 9, during the research period of the DMU, the average value of national total carbon emissions from 2009 to 2018 generally increased first and then decreased, which has a great relationship with the country paying more attention to environmental factors this year. All regions should focus more on agricultural ecological production and green development [33]. China has shown a trend of continuous emission reduction. In 2015 and 2018, the emission value was less than the target value and achieved a carbon balance.

Non-point source pollution. As shown in Figure 9, the emission reduction pressure for agricultural film residues is high and consistent with the analysis of the factor drivers, which require significant reductions except for 2018. The stress of reducing pesticide residues is low. The sample analysis period is less than the target value, related to the nation attaching great importance to pesticide residues and ensuring food safety. The loss of chemical fertilizer is relatively sound, and the pressure of reducing emissions is relatively tiny.

## 7. Conclusions

### 7.1. Conclusions

In this paper, we constructed an EBM-super-ML index model to measure the AEE of 30 regions in China under the strong disposability constraints of unexpected output and used the geographic detector method to measure the driving factors and the interaction of significant factors of eco-efficiency. The main conclusions of the research are as follows.

Firstly, this paper measures the ecological efficiency of China’s agriculture, the result shows that the mean value of AEE fluctuates around 1 overall, but fluctuates greatly in 2016 and 2017, and returns to stability in 2018. Among them, the overall efficiency (MI) fluctuated greatly in 2016 and declined in the following two years, and the main reason for this phenomenon is that technological changes (TC) showed a large technological regression in 2017. In general, the economically developed eastern region has higher technological efficiency (EC) and there is higher technological progress (TC) in the western region.

Secondly, this paper analyzes the drivers of overall effectiveness (MI). Among them, the analysis of single-factor driving factors showed that capital input, total carbon emissions, labor input, agricultural film residues, fertilizer use, and pesticide residues were the main drivers of agricultural ecological benefits, and the driving forces were 0.43, 0.37, 0.34, 0.31, 0.28, and 0.20, respectively. The interaction analysis shows that the two-factor driving effect shows a gradual trend of strengthening. Labor and capital inputs, fertilizer use, fertilizer and pesticide residues, agricultural film residues, and carbon emissions all contribute to the growth of AEE. At the same time, labor use factors significantly affect the impact of pesticides, agricultural films and carbon emissions on AEE. Capital investment significantly affects the impact of pesticide residues, agricultural film residues and carbon emissions on AEE. Therefore, rational allocation of labor resources and capital input plays an important role in reducing undesired output and improving agricultural ecological efficiency.

Thirdly, we analyze the improvement potential of AEE in China. Most scholars believe that inefficiency is a major factor in measuring the potential for improvement in AEE. The calculation results show that the proportion of agricultural ecological inefficient units in China is 0.07, mainly from the western region. The mean output inefficiency was 0.0515, of which the mean average inefficiency in seven regions exceeded 0.05, which can appropriately reduce the expected yield and reduce the undesired output to improve ecological efficiency under the constraints of environmental indicators. The average inefficiency of input factors was 0.0723, and the average value of nine regions exceeded 0.05, so adjusting the input structure of agricultural production and improving the efficiency of resource allocation between regions played an important role in improving ecological efficiency.

Fourthly, the paper provides a relaxation analysis of input and output factors. The results show that among the input factors, the intensive utilization potential of labor is the largest, followed by the input of land factors. To a certain extent, the input of agricultural machinery is not conducive to improving the ecological efficiency of agriculture. The actual value of the expected output is much higher than the target value, and the degree of relaxation is increasing year by year. Among the unexpected output index, the greatest degree of relaxation was in agricultural film. The recycling and treatment of agricultural film should be strengthened.

### 7.2. Discussion

Based on the above conclusions, we can make some suggestions on the following aspects. First, the improvement of Chinese agricultural economic efficiency mainly depends on the input of production factors [34]. However, under the constraints of environmental indicators, this extensive farming method is not conducive to the sustainable development of agriculture. Therefore, adjusting the input structure of agricultural production factors, especially the proportion of labor input, can not only adjust the proportion of fossil products, but also reduce undesired agricultural output and improve the level of sustainable agricultural development.

Second, we should also take into account the balanced development between regions, and the analysis results of the improvement potential show that in the economically developed eastern region, it is mainly manifested in the inefficiency of input factors, especially the degree of agricultural mechanization, representing the level of modern agricultural development which has adversely affected the improvement of ecological efficiency. Therefore, strengthening the development of agricultural service industries, such as agricultural cooperative economic organizations, can improve not only the efficiency of agricultural machinery, but also the efficiency of resource allocation between regions, which plays an important role in improving the ecological efficiency of China’s agriculture.

Third, the large use of agricultural film in the western region is the main cause of non-point source pollution. Therefore, promoting environmentally friendly agricultural technology innovation, using degradable agricultural film and pesticide packaging, applying bio-organic fertilizer or planting green manure instead of chemical fertilizer, can significantly reduce the level of non-point source pollution.

Fourth, economically developed regions pay more attention to green agricultural production [29] within the selected indicators and research scope, and the carbon emission level is significantly lower than that of less developed regions, which has good reference significance for regions with greater carbon emission reduction pressure.

### 7.3. Limitations and Future Research Directions

Due to space constraints, this study only analyzed agricultural eco-efficiency and its improvement potential. In the next article, we will further analyze the threshold effect and mediating effect of labor, machinery, and other factors with greater influence, and analyze the heterogeneity and spatial effect of eco-efficiency.

## Figures and Tables

**Table 1:** The type of interaction of two independent variables on the dependent variable.

Basis of Judgment	Interaction
*q*(*x*_1_∩*x*_2_) < Min(*q*(*x*_1_), *q*(*x*_2_))	Non-linear weakening
Min(*q*(*x*_1_), *q*(*x*_2_) < *q*(*x*_1_∩*x*_2_) < Max(*q*(*x*_1_), *q*(*x*_2_))	Single non-linear enhancement
*q*(*x*_1_∩*x*_2_) < Max(*q*(*x*_1_), *q*(*x*_2_))	Double enhancement
*q*(*x*_1_∩*x*_2_) = *q*(*x*_1_) + *q*(*x*_2_)	Independence
*q*(*x*_1_∩*x*_2_) > *q*(*x*_1_) + *q*(*x*_2_)	Non-linear enhancement

**Table 2:** Evaluation index and explanation of AEE.

Index	Indicator Category	Index Selection	Variables and Description
Input	Labor	Primary industry labor force	People engaged in agriculture, forestry, animal husbandry, and fishery (Ten thousand people)
	Land	The total area sown to crops	The total sown area of various crops (Ten thousand mu)
	Capital	Agricultural intermediate consumption	The value of goods and services consumed in agricultural production and operation (One hundred million yuan)
	Technology	Total power of agricultural machinery	Farming, forestry, animal husbandry, fisheries of all kinds of power machinery power sum (Kilowatts)
Output	Expected Output	Agricultural Production	∑(The total output of an agricultural product in the current year × the production price of that agricultural product) (One hundred million yuan)
	Unexpected output	Non-point source pollution	Fertilizer × Loss coefficient (Million Tons)
			Pesticide × Loss coefficient (Ton)
			Agricultural Film × Residual rate (Ton)
		Carbon Emission	Planting, Breeding, Fishery comprehensive index (Million Tons)

**Table 3:** Descriptive statistics of input–output indicators from 2009 to 2018.

Index		Max	Min	Median	Mean	Var
input	labor	1981.30	9.10	576.75	733.49	0.72
	land	138,532.90	263.30	15,183.75	20,743.87	1.19
	capital	4366.70	48.70	1139.80	1291.02	0.72
	technology	13353.00	94.00	2536.30	3318.74	0.88
output	fertilizer	465.47	4.75	119.11	125.06	0.76
	pesticide	84,521.50	892.00	28,212.25	28,901.66	0.75
	agrfilm	32,296.50	107.00	6060.90	8038.75	0.83
	CO_2_	65,389.51	84.84	14,876.24	19,293.99	0.83
	chanzhi	5272.50	100.80	1670.55	1842.79	0.69

**Table 4:** Driver setting and its discrete classification results.

Index	Affecting Factors			Classification	
	Driving Factors	Variable Declaration	Symbolic Representation	Number	Categories Method
input	labor	people engaged in agriculture	*x*_1_	4	quantile
	technology	total power of agricultural machinery	*x*_2_	4	
	land	the total area sown to crops	*x*_3_	5	
	capital	Agricultural intermediate consumption	*x*_4_	5	
		fertilizer consumption	*x*_5_	5	
		agricultural diesel consumption	*x*_6_	5	
		pesticide consumption	*x*_7_	3	
		agricultural film consumption	*x*_8_	5	
output	expect output	agricultural production	*x*_9_	4	
	unexpected output	fertilizer loss	*x*_10_	4	
		pesticide loss	*x*_11_	4	
		agricultural film residual	*x*_12_	7	
		carbon emission	*x*_13_	7	

**Table 5:** Influencing factors and driving forces of spatial differentiation of AEE.

Driving Factor	2010	Order	2012	Order	2014	Order	2016	Order	2018	Order	Mean
*x*_1_	0.334 ** (0.042)	3	0.385 ** (0.021)	2	0.192 (0.234)	8	0.378 ** (0.021)	2	0.431 *** (0.008)	3	0.34
*x*_2_	0.099 (0.500)	9	0.075 (0.666)	12	0.179 (0.283)	9	0.143 (0.430)	9	0.245 (0.122)	8	0.15
*x*_3_	0.092 (0.688)	10	0.210 (0.289)	6	0.280 (0.155)	4	0.173 (0.390)	8	0.164 (0.418)	12	0.18
*x*_4_	0.297 (0.132)	4	0.384 * (0.051)	3	0.473 ** (0.016)	1	0.435 ** (0.027)	1	0.553 *** (0.004)	1	0.43
*x*_5_	0.213 (0.281)	5	0.259 (0.188)	4	0.300 (0.127)	2	0.293 (0.136)	5	0.339 * (0.086)	4	0.28
*x*_6_	0.057 (0.918)	11	0.076 (0.758)	11	0.229 (0.246)	5	0.194 (0.331)	4	0.310 (0.116)	9	0.17
*x*_7_	0.011 (0.861)	13	0.051 (0.519)	13	0.133 (0.196)	12	0.087 (0.336)	13	0.260 ** (0.043)	5	0.11
*x*_8_	0.195 (0.326)	6	0.203 (0.306)	7	0.171 (0.395)	10	0.101 (0.650)	11	0.163 (0.421)	13	0.17
*x*_9_	0.057 (0.786)	12	0.115 (0.445)	10	0.210 (0.219)	6	0.207 (0.235)	7	0.252 (0.114)	6	0.17
*x*_10_	0.185 (0.220)	7	0.125 (0.384)	9	0.104 (0.475)	13	0.095 (0.501)	12	0.229 (0.138)	11	0.15
*x*_11_	0.104 (0.524)	8	0.153 (0.240)	8	0.208 (0.112)	7	0.280 ** (0.046)	6	0.250 * (0.069)	7	0.20
*x*_12_	0.305 (0.426)	2	0.234 (0.486)	5	0.165 (0.701)	11	0.344 (0.178)	3	0.479 * (0.070)	2	0.31
*x*_13_	0.560 ** (0.042)	1	0.673 *** (0.004)	1	0.288 (0.501)	3	0.107 (0.930)	10	0.236 (0.635)	10	0.37

Note: The q statistic is used to measure the main influence factor of the dependent variable *Y*, and the decisive power of the explanatory variable *X*. In this table, *p* values in parentheses are used to test whether the q value is significant. *, **, *** indicate significance at the 10%, 5%, and 1% levels, respectively.

**Table 6:** Interactive detection results of spatial differentiation of AEE.

Interaction Factor	2010		2012		2014		2016		2018	
	q	Type	q	Type	q	Type	q	Type	q	Type
*x*_1_∩*x*_4_	0.635	NE	0.662	NE	0.595	NE	0.703	DE	0.656	DE
*x*_1_∩*x*_5_	0.84	NE	0.906	NE	0.891	NE	0.864	NE	0.849	DE
*x*_1_∩*x*_11_	0.774	NE	0.792	NE	0.718	NE	0.64	NE	0.816	NE
*x*_1_∩*x*_12_	0.99	NE	0.825	NE	0.832	NE	0.678	NE	0.858	NE
*x*_1_∩*x*_13_	0.89	NE	0.868	NE	0.887	NE	0.864	NE	0.736	NE
*x*_4_∩*x*_5_	0.861	NE	0.791	NE	0.814	NE	0.821	DE	0.761	DE
*x*_4_∩*x*_11_	0.688	NE	0.47	NE	0.615	NE	0.838	NE	0.723	NE
*x*_4_∩*x*_12_	0.78	NE	0.709	NE	0.959	NE	0.878	NE	0.848	NE
*x*_4_∩*x*_13_	0.834	NE	0.753	NE	0.817	NE	0.686	NE	0.551	NE
*x*_5_∩*x*_11_	0.746	NE	0.577	DE	0.567	DE	0.495	DE	0.65	DE
*x*_5_∩*x*_12_	0.852	NE	0.83	NE	0.76	DE	0.709	NE	0.717	DE
*x*_5_∩*x*_13_	0.684	NE	0.685	NE	0.79	NE	0.768	NE	0.712	DE
*x*_11_∩*x*_12_	0.343	NE	0.578	DE	0.589	DE	0.466	NE	0.366	NE
*x*_11_∩*x*_13_	0.66	NE	0.561	NE	0.489	NE	0.394	NE	0.347	NE
*x*_12_∩*x*_13_	0.889	NE	0.774	NE	0.806	NE	0.736	NE	0.517	NE

Note: NE is a non-linear enhancement, and DE is a two-factor enhancement.

## Data Availability

Not applicable.
